# Multispecies Q Fever Outbreak in a Mixed Dairy Goat and Cattle Farm Based on a New Bovine-Associated Genotype of *Coxiella burnetii*

**DOI:** 10.3390/vetsci8110252

**Published:** 2021-10-26

**Authors:** Benjamin U. Bauer, Michael R. Knittler, T. Louise Herms, Dimitrios Frangoulidis, Svea Matthiesen, Dennis Tappe, Martin Runge, Martin Ganter

**Affiliations:** 1 Clinic for Swine and Small Ruminants, Forensic Medicine and Ambulatory Service, University of Veterinary Medicine Hannover, Foundation, Bischofsholer Damm 15, 30173 Hannover, Germany; martin.ganter@tiho-hannover.de; 2 Institute of Immunology, Friedrich-Loeffler-Institut, Suedufer 10, 17493 Greifswald, Isle of Riems, Germany; michael.knittler@fli.de (M.R.K.); svea.matthiesen@fli.de (S.M.); 3 Lower Saxony State Office for Consumer Protection and Food Safety (LAVES), Food and Veterinary Institute Braunschweig/Hannover, Eintrachtweg 17, 30173 Hannover, Germany; louise.pruefer@laves.niedersachsen.de (T.L.H.); martin.runge@laves.niedersachsen.de (M.R.); 4 Bundeswehr Institute of Microbiology, Neuherbergstraße 11, 80937 Munich, Germany; dimitriosfrangoulidis@bundeswehr.org; 5 Bundeswehr Medical Service Headquarters VI-2, Medical Intelligence & Information (MI2), Dachauer Straße 128, 80637 Munich, Germany; 6 Bernhard Nocht Institute for Tropical Medicine, Bernhard-Nocht-Straße 74, 20359 Hamburg, Germany; tappe@bnitm.de

**Keywords:** *Coxiella burnetii*, cat, cattle, dog, goat, MLVA/VNTR, phase-specific serology, One Health, zoonosis, 17ß-estradiol

## Abstract

A Q fever outbreak on a dairy goat and cattle farm was investigated with regard to the One Health concept. Serum samples and vaginal swabs from goats with different reproductive statuses were collected. Cows, cats, and a dog were investigated with the same sample matrix. The farmer’s family was examined by serum samples. Ruminant sera were analyzed with two phase-specific enzyme-linked immunoassays (ELISAs). Dominant immunoglobulin G (IgG) phase II levels reflected current infections in goats. The cows had high IgG phase I and II levels indicating ongoing infections. Feline, canine, and human sera tested positive by indirect fluorescent antibody test (IFAT). Animal vaginal swabs were analyzed by qPCR to detect *C. burnetii*, and almost all tested positive. A new cattle-associated *C. burnetii* genotype C16 was identified by the Multiple-Locus Variable-number tandem repeat Analysis (MLVA/VNTR) from ruminant samples. Additionally, a possible influence of 17ß-estradiol on *C. burnetii* antibody response was evaluated in goat sera. Goats in early/mid-pregnancy had significantly lower levels of phase-specific IgGs and 17ß-estradiol than goats in late pregnancy. We conclude that the cattle herd may have transmitted *C. burnetii* to the pregnant goat herd, resulting in a Q fever outbreak with one acute human case. The influence of placentation and maternal pregnancy hormones during pregnancy on the immune response is discussed.

## 1. Introduction

*Coxiella burnetii* is a globally occurring zoonotic pathogen. More than 30 different animal species are susceptible to this obligate intracellular bacterium in Europe [1]. Ruminants are the main reservoir for *C. burnetii,* which replicates in the trophoblasts of the allantochorion and placentomes [2,3]. Infected animals release enormous amounts of bacteria during abortion or normal parturition through birth products [4,5]. A *C. burnetii* infection can cause different reproductive disorders in ruminants, such as placental retention and endometritis in cattle, and endemic abortions, stillbirth, or the birth of weak offspring in goats and sheep [6,7]. 

Humans become infected easily by inhalation of contaminated aerosols, and around 40% of infected individuals show flu-like symptoms, including fever and headache, atypical pneumonia, and acute hepatitis. Only a minority acquire long-term health issues such as chronic Q fever endocarditis [8]. Small ruminants have been responsible for several large and small-scale human Q fever epidemics across Europe in recent decades [4,9,10], unlike cattle, which have caused only a limited number of human infections [4,9]. Although rarely, dogs and cats can also transmit *C. burnetii* to people [11,12,13]. 

The transmission from animals to humans has been widely studied since the discovery of *C. burnetii*. Still, information on the transmission among different animal species is scarce even though the circulation of *C. burnetii* in the animal population is a permanent hazard for people. Studies about risk factors focused on the influence of other animals and showed conflicting results regarding the role of cattle and cats as potential sources of infection for dairy goats [14,15]. 

Genotypic characterization of different *C. burnetii* isolates may give clarity to the complexity of Q fever epidemiology. The Multi-Locus Variable-number tandem repeats Analysis (MLVA/VNTR), first described in 2006 [16,17], is widely used for molecular typing of *C. burnetii* from animal and environmental samples [18,19,20]. Using this method, several genotypes were characterized within the ruminant population, and co-circulation of different genotypes within the same farm was reported [19,20,21]. In the Netherlands, outbreak investigations determined a dominant genotype on different small ruminant farms, indicating a possible transmission from one farm unit to the next [22]. Moreover, one genotype can circulate among cattle, goats, and sheep on the same premises [23]. However, this pathogen’s transmission between different species on the same farm is still poorly understood [24]. Recently, three main clusters of *C. burnetii* MLVA-genotypes were identified: Cluster A and B were mainly associated with small ruminants, and cluster C was primarily found in samples from cattle herds [20,24]. Consequently, detection of an A genotype in the cattle population has been interpreted as a potential transmission from small ruminants [24]. The opposite interpretation applies to C genotypes determined from sheep and goats [23]. The final proof of these observations is mostly missing due to the retrospective view of many molecular studies. 

A particular characteristic of *C. burnetii* is its antigenic phase variation of phase I (PhI) and phase II (PhII), which correlate with smooth and rough lipopolysaccharide changes [25,26]. IgM’s and IgG’s immune response to these phase variants has been used to characterize Q fever infections in humans by applying the indirect fluorescent antibody test (IFAT) as the gold standard [8]. Anti-PhII isotypes are predominant during primary infection, and their titers are higher than the PhI antibody titers [27]. Seropositive humans without IgM PhII titers are classified as ‘past infection’ [28], and an elevated IgG PhI titer (IgG I titer of ≥1:800) is considered as a persistent, chronic Q fever infection [8,27].

In veterinary medicine, a gold standard and standardization for the serological detection of *C. burnetii* are still missing [7,29]. Currently, the enzyme-linked immunosorbent assay (ELISA) is recommended as a serological test to diagnose *C. burnetii* in ruminants [30]. Most commercial ELISAs are based on the simultaneous detection of IgG PhI and PhII antibodies. The application of phase-specific serology has been performed in goats and cattle, and is a helpful tool to detect new infections and to analyze the disease dynamics within herds [31,32,33,34,35]. For goats, it is assumed that a rise in IgG PhII antibodies without IgG PhI antibodies indicates a recent infection, and similar levels of both antibodies imply an ongoing infection [34,35]. The detection of exclusively IgG PhI indicates that the infection occurred a long time ago [32]. In cattle, the interpretation of phase-specific antibodies is more versatile, and it is difficult to make general statements [31]. To detect *C. burnetii* antibodies in cats and dogs, the IFAT is the preferred diagnostic test [36,37].

Progesterone and estrogens are the primary female sex hormones and are involved in the control and susceptibility to bacterial infections [38]. Both steroids increase during pregnancy and alter the maternal immune response and disease pathogenesis [39]. Pregnancy levels of estrogen, in the presence of progesterone, stimulate the humoral immune response by activating the follicular helper T/B cell axis [40]. In particular, estradiol, which is produced in high concentrations by the fetoplacental unit during pregnancy, promotes the formation of antibodies against antigens [39]. Furthermore, progesterone and 17ß-estradiol have a direct impact on *C. burnetii* by inhibiting replication [41,42]. Information about the complex interaction between *C. burnetii*, sexual hormones on pregnancy levels, and immunity is lacking but may contribute to a better understanding of the pathogenesis.

The first objective of the present study was to demonstrate the complexity of a Q fever outbreak on a farm with several animal species in the sense of a One Health approach. For this purpose, dairy goats, dairy cattle, barn cats, a farm dog, and the farmer’s family were examined for the presence of a *C. burnetii* infection. To characterize the *C. burnetii* infection in the goat and cattle herd, serum samples were analyzed with phase-specific ELISAs. Moreover, the *C. burnetii* genotype isolated from bovine and caprine samples was determined by the MLVA/VNTR method to explore the potential source of infection. The second aim was to investigate the possible influence of 17ß-estradiol on the phase-specific antibody response to *C. burnetii* in goats with different pregnancy status.

## 2. Materials and Methods

### 2.1. Goats and Cattle

#### 2.1.1. Background and Sample Collection

In January 2018, a farm located in the northern German federal state of Schleswig-Holstein was affected by endemic abortion in its 360-head dairy goat herd. An aborted fetus and a placenta were sent to the local state laboratory to detect differential pathogens capable of causing abortion, such as *Brucella* spp., *Campylobacter fetus* ssp. *fetus*, *Chlamydia* spp., *C. burnetii*, *Listeria* spp., and *Salmonella* spp. The only diagnosed abortive agent was *C. burnetii* (Cq 13; VetMAX™ *C. burnetii* Absolute Quant Kit, Thermo Fisher Scientific, Dreieich, Germany). Consequently, the farmer asked the Clinic for Swine and Small Ruminants at the University of Veterinary Medicine Hannover, Foundation for help to combat the Q fever outbreak in his dairy goat herd. Further investigations started on the farm at the end of January 2018 and, at that stage, 24 multiparous goats had undergone abortion, and no live kids were born on the farm. In addition to the dairy goats, 95 dairy cattle were kept in the same barn complex with the goats, separated by a wooden wall. This wall extended from the floor to the roof, and no direct contact between goats and cattle was possible. Since November 2017, the farmer had reported reproductive disorders, including placental retention and metritis in a few dairy cows, but neither specific documentation was available nor were further measures taken to investigate the reasons. All goats were kept in one large barn in three different groups according to their reproductive statuses. One group consisted of 24 multiparous goats, which aborted within the previous three weeks. The second group contained 236 multiparous goats in their last trimester of pregnancy, and the third group comprised 100 nulliparous goats (yearlings) at early/mid-pregnancy stage. These groups were separated by metal fences (1.2 m high), and direct contact through the metal fences was possible. Moreover, all goats were kept under one roof, and thus lived in the same ambient air. The three groups were sampled for serum (KABE, Nümbrecht-Elsenroth, Germany) and vaginal swabs (Sarstedt, Nümbrecht, Germany) in January 2018 as follows: all 24 aborting goats, 30 pregnant multiparous goats, and 20 yearlings. Animals from the two latter groups were randomly selected. The kidding period of the goats took place from January 2018 until April 2018.

Moreover, 22 dairy cows with different reproductive statuses were randomly selected and sampled using the same sample matrix as indicated above. One caprine placenta from a freshly aborting goat and one bovine placenta from a freshly calved heifer were collected on the first farm visit in January 2018 for further analysis. Due to the enormous zoonotic risk, all ruminants received a primary vaccination against *C. burnetii* according to the manufacturer’s recommendations (Coxevac^®^; CEVA Santé Animale, Libourne, France) after sample collection.

#### 2.1.2. Hormone Determination in Goats

The goats’ pregnancy status was confirmed by determining the serum progesterone values with chemiluminescence using an Immulite Progesterone Kit (Siemens Healthcare, Erlangen, Germany). Goats with >1 ng/mL were defined as pregnant [43]. In this context, the 17ß-estradiol level of the pregnant goats was examined with a radioimmunoassay using a commercially available kit (Ultra-Sensitive Estradiol RIA, Beckman Coulter, Krefeld, Germany).

#### 2.1.3. Phase-Specific Antibody Detection

Ruminant sera were examined with two phase-specific ELISAs (EUROIMMUN, Lübeck, Germany). Each phase-specific ELISA separately detects either IgG PhI or IgG PhII antibodies. These ELISAs were applied according to the manufacturer’s instructions and have been recently described in detail [44]. The test results were presented quantitatively in relative units (RU) determined by a standard curve. The classification was as follows: <16 RU: negative, ≥16 RU to <22 RU: uncertain, and ≥22 RU: positive. This classification applies to both phase-specific ELISA tests. The uncertain results were assessed as negative in the current study. The results of both phase-specific ELISAs (PhI and PhII) are presented as follows: negative result: PhI- or PhII-, positive result: PhI+ or PhII+, if both phase-specific antibodies were positive, the dominant antibody level was reported as PhI++ or PhII++.

#### 2.1.4. DNA Detection and Genotyping

DNA from the vaginal swabs and the placentas was extracted with the NucleoSpin Tissue Kit (Macherey Nagel, Düren, Germany) according to the manufacturer’s instructions using the MicroLab^®^ Star (Hamilton, Gräfelfing, Germany). Subsequently, a real-time IS*1111*-PCR (VetMAX™ *C. burnetii* Absolute Quant Kit, Thermo Fisher Scientific, Dreieich, Germany) was used to detect *C. burnetii* DNA fragments. The qPCR was performed according to the manufacturer, and cycle quantification (Cq) values of ≤ 45 were assessed as positive.

One bovine and one caprine placenta plus twelve vaginal swabs from aborting goats contained enough *C. burnetii* DNA (Cq ≤ 20) to perform the MLVA/VNTR typing method. This method was recently published in detail elsewhere [20].

### 2.2. Barn Cats and Farm Dog

#### 2.2.1. Background and Sample Collection

During several farm visits, the semi-feral barn cats and the farm dog were observed eating placentas from freshly kidded or aborted goats. Furthermore, the cats and the dog were regularly fed with raw milk from goats. In May 2018, nine cats (six ♂, three ♀) were neutered for animal welfare reasons. During this procedure, serum samples (KABE, Nümbrecht-Elsenroth, Germany) from all cats and vaginal swabs (Sarstedt, Nümbrecht, Germany) from the three queens were collected. Moreover, the female dog was included within the same sampling matrix. 

#### 2.2.2. Antibody Detection

A semiquantitative IFAT was used to detect *C. burnetii* antibodies in feline and canine serum samples. This IFAT was developed to detect *C. burnetii* antibodies in serum from cats, dogs, rodents, and birds. The test was applied according to the manufacturer (Megacor Diagnostik GmbH, Hoerbranz, Austria) with slight modifications regarding titer levels. Briefly, fluorescein-labeled anti-cat IgG and anti-dog IgG were used to detect IgG antibody-antigen complexes. All serum samples and controls were tested on microscope slides coated with PhI (Nine Mile) and PhII (Nine Mile) *C. burnetii* antigens. Serum samples were diluted 1:20, 1:40, 1:80, 1:160, and 1:320 with PBS (pH 7.2). A volume of 20 µL of each sample dilution was applied to the slide and incubated for 30 min at 37 °C. Unbound antibodies were removed by washing with PBS (pH 7.2) and aqua bidest. After drying, 20 µL of the species-specific fluorescein-conjugated antibodies were applied, and the slide was incubated for a further 30 min at 37 °C. Unbound antibodies were again removed by washing with PBS and aqua bidest. Next, a glycerine (87%) dilution (1:10) was put on the slide, followed by a coverslip. Finally, the slides were observed using UV light microscopy at 400-fold magnification. Seropositive samples were identified by the presence of fluorescence from ≥1:40, and titer levels <1:40 were classified as negative samples. This test protocol is accredited according to DIN EN ISO/IEC 17025:2005. 

#### 2.2.3. DNA Detection

Vaginal swabs from the cats and the dog were analyzed by qPCR as described above (see Section 2.1.4).

### 2.3. Farmer’s Family

The family consisted of the farmer (49 years), his wife (45 years), two sons (son C: 19 years and son R: 16 years), and one daughter (6 years). Only the adults reported clinical symptoms associated with a potential *C. burnetii* infection. The farmer suffered from fatigue with a singular loss of consciousness in May 2018 and muscular pain until spring 2019. The wife, responsible for the daily goat milking, showed flu-like symptoms (e.g., fever, limb pain) in January 2018. Because painkillers did not show any improvement, she visited the family’s physician. Treatment with doxycycline led to a fast recovery. The children did not present any flu-like symptoms during the Q fever outbreak, although they sometimes worked in the goat barn and milking parlor.

Serum samples from all family members were examined in June 2018 for IgM and IgG phase-specific antibodies by IFAT according to the manufacturer (Vircell, Granada, Spain) per the treating physician’s request. IFAT titers ≥1:24 for IgM and ≥1:64 for IgG were classified as positive, and titer levels <1:24 (IgM) and <1:64 (IgG) were identified as negative. 

### 2.4. Statistical Analysis

According to the manufacturers’ instructions, the different ELISA results within the goat groups were analyzed by ranking positive or negative outcomes. These outcomes from the ELISAs were analyzed by using Fisher’s Exact Test. Differences of *C. burnetii* amounts on vaginal swabs between the three goat groups were analyzed by one-way ANOVA. Phase-specific IgG and sex hormone levels were compared between goats in early/mid-pregnancy and late pregnancy using the *t*-test and Mann–Whitney test. Results *p* < 0.05 were considered statistically significant (GraphPad Prism 9, GraphPad Software, San Diego, CA, USA).

## 3. Results

### 3.1. Goats and Cattle

Pregnancy was confirmed by progesterone values higher than 1 ng/mL in 23 goats in late pregnancy (*n* = 30) and 16 goats at early/mid-pregnancy (*n* = 20). These animals together with the aborting goats (*n* = 24) were included in further analyses. 

**Figure 1:**
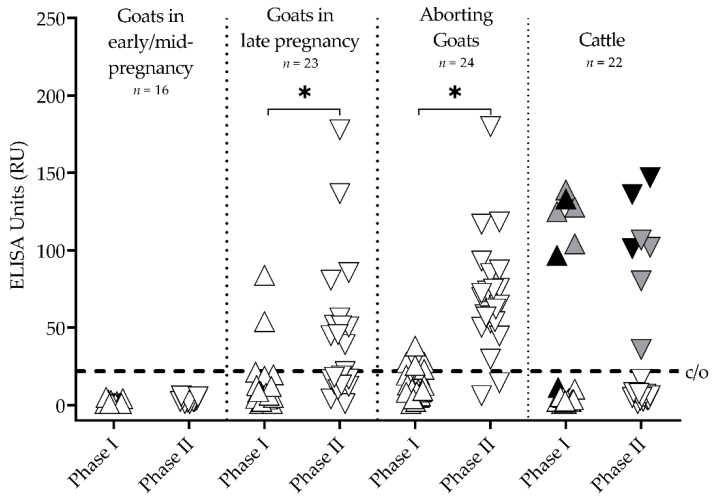
IgG antibody response against *Coxiella burnetii* of three goat groups with different reproductive status and cattle measured by two phase-specific ELISAs (Phase I △ and Phase II ▽). * Phase II ELISA detected more positive goats than Phase I (*p* < 0.05); c/o = ELISA cut-offs; colored points represent individual cattle: black = cows with IgG Phase II dominance, gray = cows with IgG Phase I dominance.

The aborting goats and the goats in late pregnancy showed a dominance of IgG PhII antibodies compared to IgG PhI (Figure 1). The IgG PhII ELISA detected, in both groups, more seropositive animals than the IgG PhI ELISA (*p* < 0.05). All pregnant yearlings were seronegative.

The IgG PhI and IgG PhII mean levels (±standard error) were significantly different between goats in early/mid-pregnancy (PhI 1.9 ± 0.3 RU, PhII 3.2 ± 0.4 RU) and late pregnancy (PhI 13.3 ± 4.0 RU, PhII 42.7 ± 9.1 RU).

In cattle, seven of 22 animals tested seropositive with the IgG PhII ELISA (Figure 1). Among these seven seropositive animals, IgG PhI antibodies were detected in six cows. In detail, three seropositive cows had higher IgG PhII antibody levels compared to IgG PhI (PhI-/PhII+, PhI+/PhII++; Figure 1 black), and four cows had higher IgG PhI titers than IgG PhII (PhI++/PhII+; Figure 1 gray).

**Figure 2:**
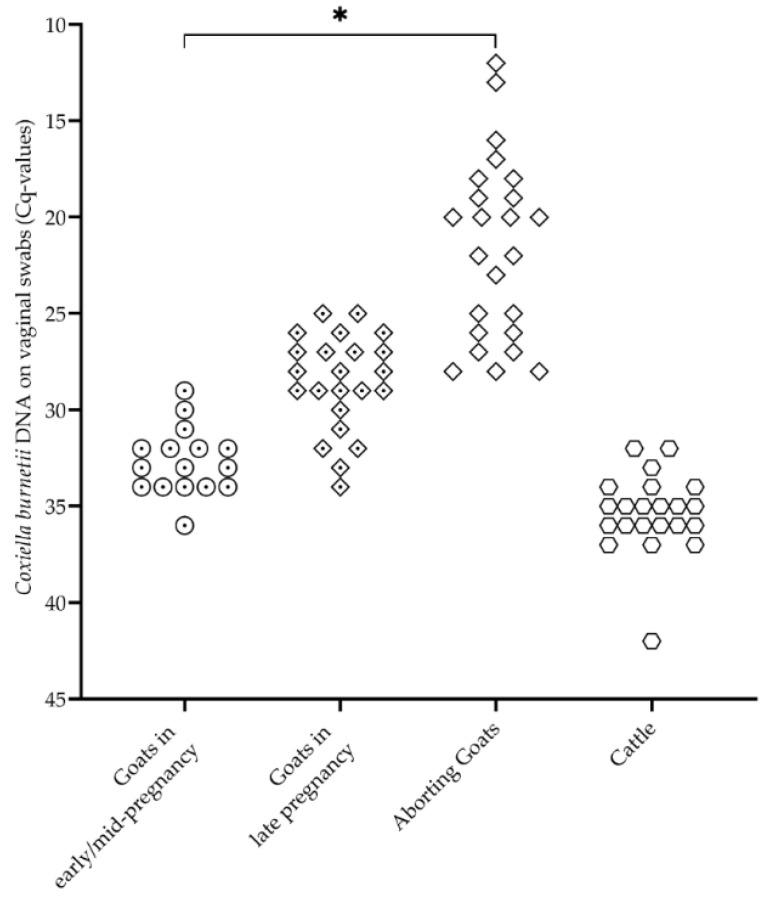
*Coxiella burnetii* DNA on vaginal swabs from three goat groups with different reproductive statuses (goats in early/mid-pregnancy ⊙ (*n* = 16), goats in late pregnancy ⟐ (*n* = 23), aborted goats ◇ (*n* = 24)) and cattle ⎔ (*n* = 22). Each point represents the qPCR result (Cq-value) of an animal. * Significant differences between the three groups of goats (*p* < 0.05).

All vaginal swabs from the goats and cattle tested positive for *C. burnetii* DNA (Figure 2). The amount of pathogen on the vaginal swabs detected by the semiquantitative qPCR differed between the three goat groups (*p* < 0.05). Furthermore, the placenta from an aborting goat revealed a Cq-value of nine, and the placenta from a heifer also tested *C. burnetii* positive (Cq 20).

**Figure 3:**
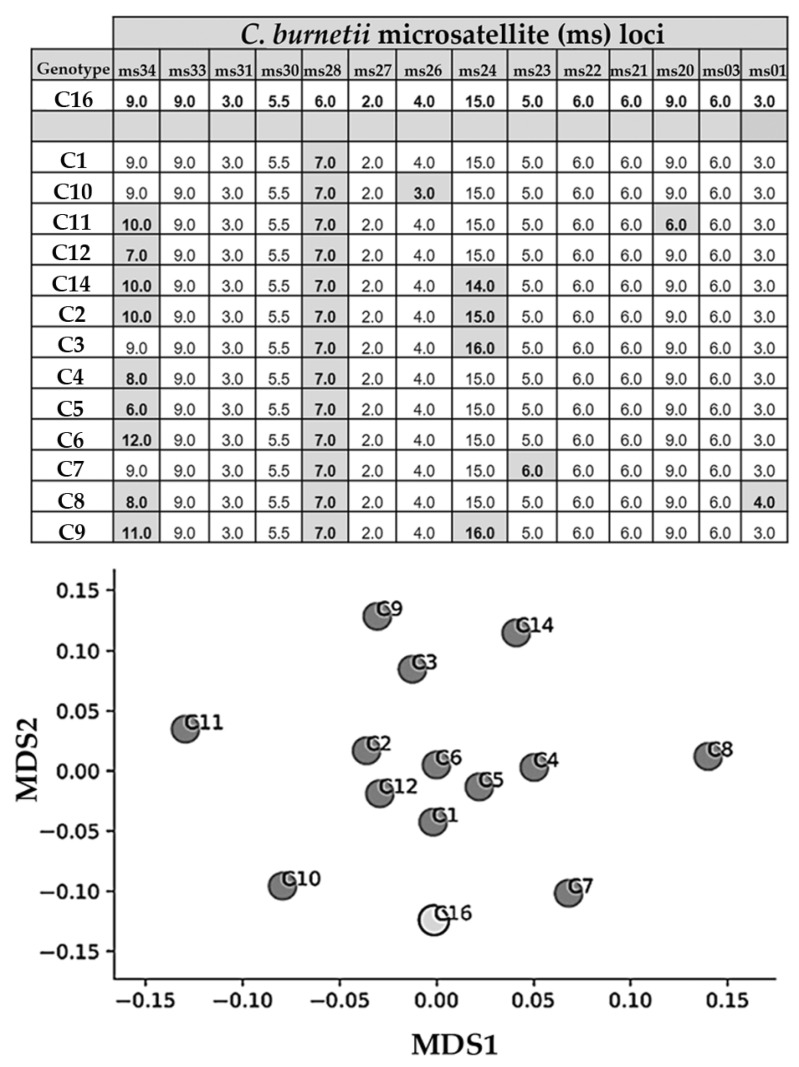
Result-table (top) from the CoxBase-MLVA-query when entering the genotyping profile from this study. For the first time, C16 was identified in bovine and caprine samples, and the next related MLVA-genotypes are C1, C7, and C10. Findings of the performed MLVA/VNTR analyses are depicted for different microsatellites’ (ms) loci (ms34, 33, 31, 30, 28, 27, 26, 24, 23, 22, 21, 20, 03, and 01). Loci from other known *Coxiella* genotypes, which do not match with the loci of the genotype C16, have a gray background. The multi-dimensional scaling (MDS) plot on the bottom created with Python depicts Jaccard similarity coefficients of the performed microsatellites analysis of the different *Coxiella burnetii* genotypes.

Application of MLVA-genotyping revealed a pattern with the 14 markers, which was transferred to a recently developed and published *Coxiella*-specific web-based genotyping database, CoxBase [45]. When comparing the typing pattern with more than 400 MLVA-typing entries, a new genotype inside the ‘C’-cluster (cattle-associated) was identified and named C16. For the first time, this cattle-associated genotype C16 was determined from the bovine and caprine placentas and the twelve caprine vaginal swabs. This new C16 genotype is closely related to the MLVA-genotypes C1, C7, and C10 (Figure 3), which were identified in a previous study [20].

**Figure 4:**
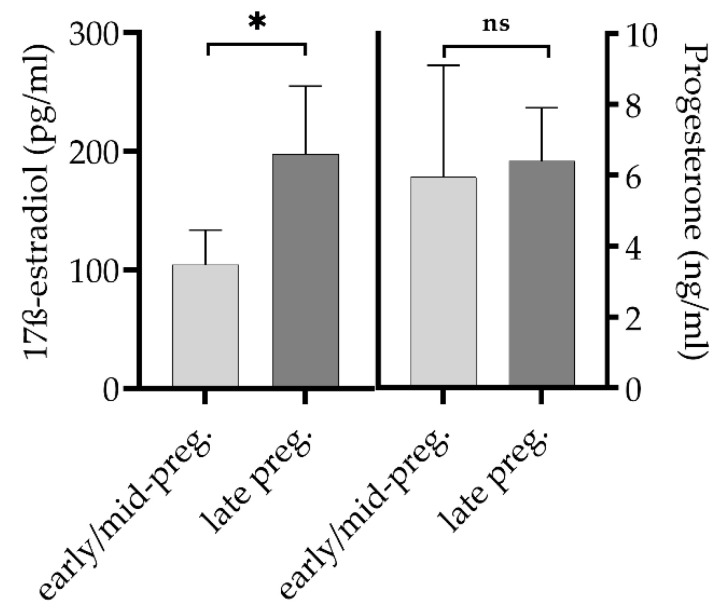
17ß-estradiol (mean, 95% confidence interval) and progesterone (median, 95% confidence interval) levels of goats in early/mid-pregnancy (*n* = 16) and in late pregnancy (*n* = 23). Difference of 17ß-estradiol levels between both groups is significant (* *p* = 0.01). ns = not significant.

The 17ß-estradiol values were significantly different between goats in early/mid-pregnancy and late pregnancy, but the progesterone concentrations of both groups were at equal levels (*p* > 0.05) (Figure 4).

### 3.2. Barn Cats and Farm Dog

Three tomcats had *C. burnetii* antibody titers of 1:160 in the IFAT and one queen, which had previously given birth to three kittens, had a titer of 1:320. The other five cats were seronegative. All feline vaginal swabs tested *C. burnetii* positive by qPCR (Cq 31, Cq 32, Cq 35). The farm dog was seropositive (1:40), but its vaginal swab showed no reaction in the qPCR. 

### 3.3. Farmer’s Family

In June 2018, the following IgM and IgG titers were detected in the family members by IFAT: father: IgG PhII 1:128; son R: IgG PhII 1:64; daughter: IgG PhI and PhII: 1:64; son C: negative. The mother showed a low IgM PhI titer (1:24) and an IgG PhII titer of 1:512 with an IgG PhI titer of 1:128.

## 4. Discussion

**Figure 5:**
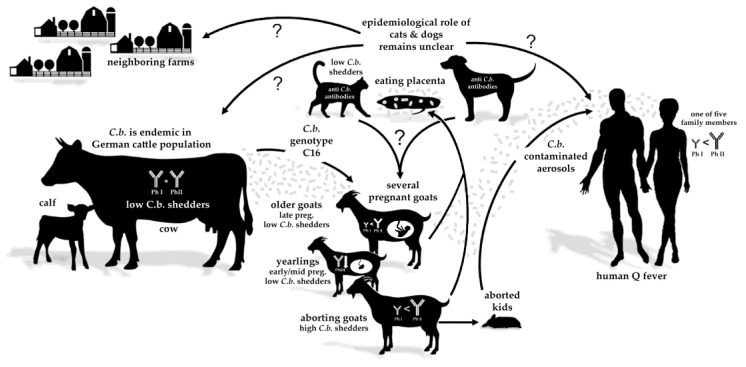
Possible transmission routes of *Coxiella burnetii* (*C.b.*), genotype C16, on a dairy farm with different species (including humans) living on the same farm. The epidemiological role of cats and dogs (eating caprine placentas) in transmitting *C. burnetii* to other species, humans, and neighboring farms is uncertain.

The present study describes an acute Q fever outbreak on a mixed dairy goat and cattle farm involving different animal species and the farmer’s family (Figure 5). Generally, *C. burnetii* circulation within a farm is poorly understood [24]. Therefore, serological and molecular investigations, including of all susceptible farm animal species and humans, are essential for understanding the dynamics of Q fever outbreaks and are in line with the One Health concept.

This was the first time that the cattle-associated genotype C16 was identified from caprine and bovine samples originating from the same farm. Cluster C is usually associated with the cattle population but, in rare cases, C1, C7, and C14 genotypes have also been identified in samples from goats [20,23,24]. In detail, the C7 genotype was detected on a farm with goats and cattle [23], but no information was provided about the presence of cattle when the C1 and C14 genotypes were described in samples from goats [20,24]. Nevertheless, *C. burnetii* genotypes from the C Cluster can circulate among ruminant species on the same farm. Comparison with other studies is hampered because of the different numbers of markers used for the MLVA/VNTR method. Efforts to standardize the MLVA/VNTR method, including the nomenclature, were recently published for a broad audience on the CoxBase platform (https://coxbase.q-gaps.de; accessed on 15 September 2021) and were also used for the analysis of genotyping results in this study [45].

Infected cattle may easily transmit the *C. burnetii* genotype C to pregnant goats. During pregnancy, goats are highly susceptible to a *C. burnetii* infection and, therefore, are at risk of acquiring Q fever [46]. The serological findings support our supposition. Most seropositive cows showed high IgG PhI and PhII antibodies, indicating an ongoing infection with *C. burnetii* [31,47]. The proportion of 30% seropositive cows in the current dairy cattle herd was at least twice as high as reported in other German cattle herds [31]. The goat flock’s phase-specific serology revealed an acute Q fever infection by the dominance of IgG PhII and low IgG PhI antibodies [34,35]. Our interpretation is additionally supported by detecting low Cq values (≤20) in the vaginal swabs and placentas of aborting goats. Such high quantities of *C. burnetii* were regularly found in goats suffering from acute Q fever [2,48]. Consequently, infected cattle herds must be considered a potential source of *C. burnetii,* and these herds may transmit the pathogen to other animal species [23,49]. This may play a crucial role in the complex *C. burnetii* epidemiology due to the endemic distribution of *C. burnetii* in the German dairy cattle population [50]. Hence, a high cattle density can be a risk factor for dairy goats to acquire a *C. burnetii* infection [15].

The serological results of the farmer’s family from June 2018 indicate that the mother acquired a recent *C. burnetii* infection, possibly during the Q fever outbreak in the dairy goat herd. She conducted high-risk activities such as daily milking and obstetrics [28]. Additionally, her medical history with flu-like symptoms and cure after doxycycline treatment supports this assumption. The other family members probably acquired a *C. burnetii* infection before the Q fever outbreak in the goat herd in January 2018, but the source for their infection remains unclear.

In the present case, it was observed that the cats and the dog ate placentas from kidding and aborting goats and were fed with contaminated raw goat milk. One seropositive queen recently gave birth to three kittens, and *C. burnetii* DNA (Cq 32) was detected from the vaginal swab of this cat. In this context, it should be taken into account that vaginal swabs collected in a contaminated environment have limited diagnostic value due to the risk of sample contamination [2]. Nevertheless, it may be possible that *C. burnetii* was excreted by this queen during parturition. Therefore, *C. burnetii*-infected cats may act as a potential source of infection for other animals or humans. Cats on dairy goat farms increase the risk for goats and people living and working on the farms to be *C. burnetii* seropositive [15,28], and cases of cat-associated Q fever outbreaks in humans have been reported previously [12,13]. Moreover, the presence of dogs in goat barns also increases the probability of goats being *C. burnetii* seropositive [15]. Infected cats and dogs may disseminate *C. burnetii* between farms, but this has not yet been proven. Hence, companion animals’ role in the Q fever epidemiology on farms remains uncertain and needs further investigation.

The three groups of goats with different reproductive statuses showed distinct intensities in their IgG response to *C. burnetii* infections. Thus, our observation suggests that the timing of gestation of the goats most likely impacts the humoral immune response against *C. burnetii*. Indeed, comparing multiparous goats in late pregnancy with yearlings in early/mid-pregnancy revealed significant differences between the levels of the gestation hormone 17ß-estradiol and the levels of IgG PhI/II (Figure 1 and Figure 4). Most interestingly, there is evidence that estrogen, which continuously increases from early to late pregnancy, has a direct or indirect stimulating effect on CD4+ T and B cells [51,52,53]. In combination with our observations, this suggests a putative immune-stimulating estrogen-effect on IgG production against *C. burnetii* in late/advanced pregnancy. The hypothesis about the impact of sexual hormones on the immune response to *C. burnetii* in goats is supported by previous findings from Roest et al. [34].

Despite different placental interhemal barriers, which are syndesmochorial for ruminants, *C. burnetii* shows a pronounced affinity for the placental tissue during host infection [54]. In mammals, the oxygen concentrations at the site of embryo implantation and during the placenta’s initial formation are reduced [55,56,57]. It can also be assumed that in small ruminants, oxygen tension increases from early placenta epithelichorealis to the later fetomaternal syncytium [58]. Under hypoxic environmental conditions, *C. burnetii* displays no bacterial replication but remains fully viable, allowing persistent and efficient immune escape of the pathogen [59]. Therefore, it is tempting to assume that early-onset hypoxia in placental development affects *Coxiella*’s development/growth and, in turn, the pathogen’s visibility to the host’s immune system during early gestation. This is probably accompanied by the increasing levels of pregnancy hormones (e.g., progesterone), which influence *Coxiella*’s replication [41], and probably detection by the immune system. Based on significant differences between goats at early/mid and advanced pregnancy on IgG response and vaginal shedding (Figure 1 and Figure 2), one can assume that the sharp decrease in progesterone around day 126 of pregnancy influences the *C. burnetii* load in trophoblasts and thereby stimulates the humoral immune response at the end of pregnancy. 

Moreover, given our findings on the infected pregnant goats, it must also be considered that the fetoplacental unit is one of the immune-privileged organs in mammals (from implantation to parturition), meaning it can tolerate antigens without eliciting an immune response [60]. This crucial property of the placenta may also contribute to the differences in the anti-PhI/II-responses against *C. burnetii* observed for aborted and pregnant goats (early or late). Fetal trophoblasts have an essential function in the interaction of placenta/fetus and maternal immune system [61]. This is based on the control of leukocyte immigration and effector function. Thus, trophoblasts express both indoleamine-2, 3-dioxygenase (IDO) and Fas ligands to functionally knockdown maternal lymphocytes [62,63]. Furthermore, tolerance and immunosuppression in the placenta are controlled by uterine NK cells (uNK), dendritic cells (DCs), and regulatory T cells (Tregs) [64,65,66,67,68,69,70,71], which may also suppress the adaptive immune system of the infected mother [72]. Taken together, these known scenarios, in conjunction with our own observations, offer a possible explanation for the lack of detectable IgG PhI/II antibodies during early/mid-pregnancy in *C. burnetii*-infected small ruminants. Clearly, further intensive immunological studies are needed to analyze the interesting and crucial aspects of *Coxiella* infection during placenta development and gestation.

## 5. Conclusions

Based on the detection of a novel cattle-associated *C. burnetii* C16 MLVA-genotype in samples from cattle and goats, and the phase-specific antibody patterns, we hypothesize direct transmission of *C. burnetii* from an infected dairy cattle herd to a nearby pregnant dairy goat herd. Therefore, we consider cattle herds as potential reservoirs capable of transmitting *C. burnetii* to other species on a farm. In the present study, the possible transmission from the cattle herd to the pregnant goats led to an acute Q fever outbreak in the goat herd with endemic abortion, followed by a probable acute infection of at least one family member and other animal species on the farm. Our findings on multispecies transmission of *C. burnetii* and the possible influence of 17ß-estradiol on phase-specific IgG response in pregnant goats are based only on one farm outbreak and a relative low number of samples, which limit the significance of our investigations. In the future, studies should focus on more multispecies farms to evaluate their risk potential of *C. burnetii* transmission. To clarify the influence of sexual hormones and placental development on immune response to *C. burnetii*, more intensive investigations under controlled conditions are urgently needed. According to the One Health concept, this outbreak shows that a holistic approach is necessary to manage Q fever outbreaks on farms.

## Data Availability

The data are available on request from the corresponding author.
